# Körperpsychotherapeutische Gruppentherapie für jugendliche Patientinnen mit Anorexia nervosa: Erste Ergebnisse zu Therapiebeurteilung und Körpermaßschätzungen

**DOI:** 10.1007/s00278-023-00655-9

**Published:** 2023-04-04

**Authors:** Lea Anna Graute, Ida Wessing, Anke Dalhoff

**Affiliations:** Klinik für Kinder- u. Jugendpsychiatrie, -psychosomatik und -psychotherapie, Universitätsklinikum Münster, Schmeddingstr. 50, 48149 Münster, Deutschland

**Keywords:** Konzentrative Bewegungstherapie, Körperbild, Körpermaß, Wahrnehmung, Umfragen und Fragebogen, Concentrative movement therapy, Body image, Body size, Perception, Surveys and questionnaires

## Abstract

**Hintergrund:**

Ansätze zur Behandlung der Körperbildstörung, Kernsymptom der Anorexia nervosa (AN), fokussieren unterschiedliche Komponenten (perzeptiv, kognitiv-affektiv). Auf Basis der Beobachtung massiver Störungen der ganzheitlichen Körperwahrnehmung bei jugendlichen AN-Patientinnen wurde eine störungsspezifische körperpsychotherapeutische Gruppentherapie entwickelt, die gezielt Aspekte des Körpererlebens integriert.

**Ziel der Arbeit:**

Evaluation und Untersuchung von Zusammenhängen mit Veränderungen des perzeptiven Körperbildes.

**Material und Methoden:**

Am Ende der Gruppenteilnahme füllten 52 AN-Patentinnen (Alter: *Mittelwert* = 15,35 Jahre) den Therapiebeurteilungsfragebogen zur Konzentrativen Bewegungstherapie im Gruppen-Setting (TBF-KBT-G) aus. Körpermaßschätzungen mithilfe des Test for Body Image Distortion in Children and Adolescents (BID) zu Beginn und Ende der Gruppenteilnahme wurden verglichen. Korrelationen wurden genutzt, um Zusammenhänge zwischen Therapiebeurteilung und Veränderung der Körpermaßschätzung zu untersuchen.

**Ergebnisse:**

Patientinnen berichteten von einer positiven Beziehung zur Therapeutin, einer positiven Gruppenatmosphäre sowie teils von positiven körperbezogenen Erfahrungen und vertieftem Selbstzugang anhand der KBT-Methode. Die Patientinnen überschätzten ihre Körpermaße, und dies bestand am Ende der Gruppenteilnahme fort. Patientinnen mit positiven körperbezogenen Erfahrungen zeigten eher eine bessere Körpermaßschätzung.

**Diskussion:**

Trotz des Fokus auf den aversiv erlebten Körper beschrieben die jugendlichen AN-Patientinnen ein positives Beziehungserleben in der Gruppe und können die KBT-Methode teilweise gut für sich nutzen. Störungen des perzeptiven Körperbildes erscheinen relativ persistent; eher mit einer Verbesserung assoziiert waren positive körperbezogene Erfahrungen. Insgesamt sind diese Ergebnisse als ermutigend zu interpretieren.

## Abstract (en)

**Background:**

Approaches to the treatment of body image distortion, a key symptom of anorexia nervosa (AN), focus on different components (perceptive, cognitive affective). The observation of massive disturbances in holistic body perception in adolescent female AN patients was the basis for the development of a disorder-specific body psychotherapeutic group therapy that specifically integrates aspects of body experience.

**Objective:**

Evaluation of a body psychotherapeutic group therapy for adolescent AN patients and analysis of possible correlations with changes in perceptive body image.

**Material and methods:**

52 female AN patients (mean age 15.35 years) filled in the therapy assessment questionnaire for concentrative movement therapy (CMT)® in a group setting (in German: TBF-KBT‑G) at the end of the group participation. Body size estimations using the test for body image distortion in children and adolescents (BID) were compared at the beginning and end of the group participation. The correlations were used to examine associations between therapy evaluation and changes in body size estimations.

**Results:**

The patients reported a positive relationship with the therapist, a positive group atmosphere, partly reported positive body-related experiences and deeper access to themselves using the CMT method. The AN patients overestimated their body size and this was unchanged on average at the end of group participation. Patients with positive body-related experiences tended to show improved body size estimation.

**Conclusion:**

Despite the focus on the aversively experienced body, adolescent female AN patients described experiencing positive relationships in the group and were partly able to take advantage of the CMT method. Disturbances of the perceptive body image appear to be relatively persistent, whereby positive body-related experiences tended to be associated with an improvement. Overall, these are encouraging results.

Körperbildstörungen sind ein Kernsymptom der Anorexia nervosa (AN) und stellen eine große therapeutische Herausforderung dar. Therapeutische Ansätze zur gezielten Behandlung sind jedoch wenig erforscht. Im Folgenden werden Ergebnisse der subjektiven Evaluation einer körperpsychotherapeutischen Gruppentherapie zur Behandlung von Körperbildstörungen bei Jugendlichen mit AN auf Grundlage der Konzentrativen Bewegungstherapie (KBT)® dargestellt.

## Hintergrund und Fragestellung

Ein Kernsymptom der Anorexia nervosa (AN) ist die Körperbildstörung, die in verschiedene Komponenten unterteilt werden kann (Thompson 2004). Die kognitiv-affektive Komponente beinhaltet die Körperunzufriedenheit und die Bewertung des eigenen Körpers mit negativen Gedanken und Gefühlen (Steinfeld et al. 2017). Kennzeichnend für die perzeptive Komponente ist die Überschätzung der eigenen Körpermaße, z. B. durch verzerrte Körperrepräsentationen (Mölbert et al. 2017). Die zentrale Bedeutung der Körperbildstörung zeigt sich in der Vorhersage einer Manifestation (Rohde et al. 2015) sowie prädiktiv in höheren Rückfallraten (Keel et al. 2005) und in schlechteren Langzeitverläufen (Boehm et al. 2016). Trotz dieser zentralen Bedeutung ist eine gezielte Behandlung der Körperbildstörung bei AN nicht selbstverständlich und wissenschaftlich wenig untersucht (Cascino et al. 2019).

Bisher am besten untersucht sind kognitiv-behaviorale Ansätze der Körperbildtherapie, z. B. Vocks et al. (2018). Der Fokus liegt auf der kognitiv-affektiven Komponente mit der Annahme, dass dysfunktionale Gedanken in Form einer Überbeschäftigung mit dem Essen, der Figur und dem Gewicht ausschlaggebend sind (Legenbauer 2022). Diese können über kognitive Techniken identifiziert und modifiziert werden. Hinweise auf die Wirksamkeit äußern sich in einer Verringerung dysfunktionaler Kognitionen, wie z. B. Figur- und Gewichtssorgen (Legenbauer et al. 2011; Vocks et al. 2018). Des Weiteren werden Körperexpositionen angewendet, mit der Annahme, dass der Körper aufgrund negativer Gefühle, v. a. Angst, übermäßig kontrolliert und vermieden wird, z. B. durch Tragen weiter Kleidung (Biney et al. 2021). Ziel ist, mithilfe von Spiegelkonfrontationen körperbezogene Angst abzubauen und verzerrte Körperwahrnehmungen zu korrigieren (Tanck et al. 2022). Bei Jugendlichen zeigt sich die Wirksamkeit von Spiegelexpositionen in einer Verbesserung von Gewichtssorgen, körperbezogenem Vermeidungsverhalten und Ängsten in Bezug auf das körperliche Erscheinungsbild (Biney et al. 2021), ähnlich wie bei Erwachsenen (Morgan et al. 2014). Entsprechend den gesetzten Behandlungsfoci und Untersuchungsmethoden werden Veränderungen v. a. in den Bereichen Kognition und Verhalten erzielt.

Ein weiterer Ansatz legt den Fokus auf die perzeptive Komponente der Körperbildstörung. Artoni et al. (2021) gehen davon aus, dass sich bei PatientInnen mit langen Verläufen der AN starvationsbedingt negative Körperrepräsentationen verfestigen. Im Gruppen-Setting durchgeführte, wiederholte Körperwahrnehmungsübungen sollen die Selbstwahrnehmung stärken und mit korrekten Informationen über den Körper aktualisieren. Im Vergleich einer kognitiv orientierten stationären Behandlung erwachsener AN-PatientInnen mit und ohne Teilnahme an dieser Gruppe wiesen GruppenteilnehmerInnen eine signifikantere Verbesserung der allgemeinen und essstörungsspezifischen Psychopathologie auf. Zudem zeigten nur GruppenteilnehmerInnen eine Verbesserung im Körperunbehagen, was auf eine spezifische Wirkung auf das Körperempfinden hinweist.

Die bisher dargestellten Behandlungsansätze machen deutlich, dass unterschiedliche Komponenten der Körperbildstörung möglicherweise unterschiedliche therapeutische Zugangswege erfordern. In diesem Kontext werden im Folgenden erste Daten zur Evaluation einer körperpsychotherapeutischen Gruppentherapie, basierend auf der KBT, vorgestellt (Dalhoff 2022a). Sie richtet sich speziell an jugendliche AN-Patientinnen und legt – ähnlich wie Artoni et al. bei Erwachsenen – einen Fokus auf das perzeptive Körpererleben, geht jedoch mit einem interaktionellen Ansatz deutlich über Körperwahrnehmungsübungen hinaus.

Aus körperpsychotherapeutischer Sicht zeigen AN-Patientinnen ein komplexes Bild einer gestörten Körperwahrnehmung nicht nur bezüglich der Körpermaße, sondern auch der Körpersignale und -empfindungen; sie sind nicht mehr in der Lage, Letztere vollständig wahrzunehmen und zu interpretieren (Schreiber-Willnow 2016). Es kommt zum Verlust eines ganzheitlichen Körperselbsterlebens in verschiedene Geraden von Fragmentierung. Anorexia nervosa ist mit Schwierigkeiten, die eigenen Gefühle zu erkennen und zu verbalisieren (Alexithymie), sowie mit Schwierigkeiten in zwischenmenschlichen Beziehungen verbunden (Lukas et al. 2022).

Hieraus ergibt sich die Annahme, dass bereits ersterkrankte jugendliche AN-Patientinnen unter massiven Störungen der Körperwahrnehmung leiden. Die klinische Erfahrung unter Verwendung (halb-)standardisierter Verfahren zur Erfassung perzeptiver Körperbildstörungen (u. a. Körpermaßschätzung und Körperbild-Skulptur-Test nach Joraschky et al. 1998) bestätigt bei diesen Patientinnen im Zustand der Starvation eine stark fragmentierte Körperwahrnehmung, die eng mit Kontakt- und Beziehungsstörungen verbunden ist. Auf Basis dieser Beobachtungen wurde ein störungsspezifisches Behandlungskonzept mit unterschiedlichen Phasen entsprechend dem sich im Behandlungsverlauf verändernden Strukturniveau entwickelt (Dalhoff 2022a). Die Patientinnen werden darin unterstützt, ihre Körperwahrnehmung zu verbessern sowie einen tieferen Zugang zu sich und ihrem Körper (ohne Wertung) zu erlangen. Davon ausgehend wird interaktiv an einer Integration der Körperwahrnehmungen in das Selbst(Körper‑)Bild (perzeptive und kognitiv-affektive Komponente) gearbeitet, und das Ich-Erleben erfährt in der Gruppe eine Resonanz.

## Ziele der Arbeit

Ziele dieser Arbeit sind die Darstellung erster Ergebnisse der Evaluation einer körperpsychotherapeutischen Gruppentherapie für jugendliche AN-Patientinnen sowie die Untersuchung von Zusammenhängen der Therapiebeurteilung mit Veränderungen des perzeptiven Körperbildes. Hypothesen waren, dass die Patientinnen die Therapie positiv beurteilen, und dass ein positives Erleben der Gruppentherapie mit Verbesserungen in der Körpermaßschätzung einhergeht.

## Methoden

### Stichprobe

In die Studie eingeschlossen wurden Patientinnen mit einer restriktiven, aktiven oder atypischen AN gemäß der 10. Version der Internationalen statistischen Klassifikation der Krankheiten und verwandter Gesundheitsprobleme (ICD-10); die Patientinnen waren vom Mai 2019 bis zum November 2022 stationär behandelt worden und hatten an mindestens 8 Sitzungen der körperpsychotherapeutischen Gruppentherapie teilgenommen. Ausschlusskriterien waren wiederholte stationäre Aufenthalte und die Diagnosen Abhängigkeitssyndrom, tiefgreifende Entwicklungsstörung, Schizophrenie oder bipolare Störung. Die Diagnosestellung erfolgte durch die behandelnden TherapeutInnen anhand der diagnostischen ICD-10-Kriterien und unter Einbezug von Fragebogen zu Essstörungs- und komorbider Symptomatik (Tab. 2). Die Ethikkommission der Ärztekammer Westfalen-Lippe und der Universität Münster erteilte ein positives Ethikvotum (2019-079-f-S). Alle Patientinnen und Eltern wurden umfassend mündlich und schriftlich aufgeklärt und erklärten schriftlich ihre Teilnahmebereitschaft.

### Behandlungsablauf auf der Station

Die stationäre Behandlung erfolgte anhand eines störungsspezifischen Behandlungskonzeptes. Es wurden ein Mindestgewicht (25. Altersperzentile) und eine wöchentliche Gewichtszunahme (≥ 700 g) vereinbart.

Die multimodale Behandlung umfasste Einzel- und Gruppentherapie, Familiengespräche, Psychoedukationsgruppe, Ernährungs- und Kochgruppen sowie Gruppenaktivitäten. Patientinnen im Zustand der starken Starvation nahmen zunächst an der Körperschemagruppe (Körperwahrnehmungsübungen ohne Reflexion/Interaktion) und folgend an der körperpsychotherapeutischen Gruppentherapie teil. Parallel erhielten die Patientinnen eine körperpsychotherapeutische Einzel- und Familientherapie (Dalhoff 2022b).

### Ablauf der körperpsychotherapeutischen Gruppentherapie

Jede Sitzung beginnt und schließt mit dem Kreis und der Ermutigung, für das innere Erleben eigene Worte zu finden. Davon ausgehend regt die KBT-Therapeutin zum Explorieren im Raum, mit sich und den Anderen an. Darauf folgt ein angeleitetes Erfahrungsangebot. Durch die verschiedenen Herangehensweisen der KBT, wie z. B. Liegen, Sitzen, Stehen, Gehen (Erleben des Körpers im motorischen Ausdruck) oder Geben und Nehmen (in der Bewegung Beziehung erleben), wird zu freien Bewegungsassoziationen (Becker 2001) angeregt. Durch die konzentrative Einengung (Aufmerksamkeitsfokussierung auf interozeptive und taktile Wahrnehmung) wird das Körperbild aktiviert und erfahrbar, und dies bestimmt den „Weg vom Wahrnehmen zum Vergleichen, Erproben, Wählen, Entscheiden, Verändern und Handeln“ (Gräff 2008). Körpererinnerungen, Empfindungen und Gefühle werden (wieder)belebt, mit Erinnerungsbildern verknüpft und neue Erinnerungsbilder geschaffen. Die Patientinnen werden zum aktiven Erproben angeregt, entweder auf der Selbstebene oder in der Begegnung mit den Anderen. Anhand dieser Erfahrungen werden Beziehungsthemen, wie z. B. Nähe und Distanz, Reibung und Widerstand, Aggression und Hemmung, fokussiert. Im Erfahrungsraum mit sich und den Anderen werden Gefühle intrapsychisch und intersubjektiv spürbar. Es entsteht ein Wechselspiel von Sichzeigen, Sichverbergen, Ansehen und Gesehenwerden, verbunden mit entscheidenden Momenten von Resonanzerfahrung. Dies erzeugt ein Diskrepanzerleben (z. B. Wie wünsche ich mir, gesehen zu werden, und wie werde ich vom Anderen wahrgenommen? Wie geht es mir, wenn ich den Anderen berühre oder berührt werde?) verbunden mit intensivem Körpererleben wie z. B. Schamgefühlen, Erleben von Entgrenzung/fehlenden Körpergrenzen, dissoziativem Erleben an bestimmten Körperregionen. Die Auseinandersetzung mit diesem Körpererleben in der Gruppe fördert Einsicht in Zusammenhänge mit der anorektischen Symptomatik (psychoedukativer Anteil) und stärkt das Identitätserleben sowie Zugehörigkeitsgefühle. Dies wirkt dem Selbstverlust mit Fragmentierung entgegen.

### Erhebungsinstrumente

#### Psychopathologie

Depressive Symptome wurden mithilfe des Beck-Depressions-Inventar, 2. Auflage (BDI‑2; Hautzinger et al. 2009) erhoben und Körperunzufriedenheit mit dem Fragebogen zum Figurbewusstsein (FFB; Pook et al. 2002). Essstörungssymptome wurden bei einem Teil der Stichprobe mithilfe des Eating Disorder Inventory for Children (EDI‑C; deutsche Version; Thiels et al. 2011) erfasst und nach einer Umstellung bei dem folgenden Teil der Stichprobe mithilfe des Eating Disorder Examination – Questionnaire (EDE‑Q; Hilbert et al. 2007).

#### Körpermaßschätzung

Die Wahrnehmungsverzerrung eigener Körpermaße wurde mit dem Test for Body Image Distortion in Children and Adolescents (BID; Schneider et al. 2009) zu Beginn (T1) und zum Ende der Teilnahme an der körperpsychotherapeutischen Gruppe (T2) erfasst. Die Patientinnen schätzten 3 verschiedene Körpermaße: Umfang des Oberarms in Höhe der Achselhöhle, Umfang des Oberschenkels in Höhe des Schritts und Umfang der Taille in Höhe des Bauchnabels. Der geschätzte Umfang wurde mit einem Seil gelegt und die Länge des Seils gemessen. Anschließend wurde das Seil um die entsprechenden Körperteile gelegt und erneut gemessen. Zur Bestimmung von Indexwerten für Oberarm (BID-OA), Taille (BID-Taille) und Oberschenkel (BID-OS) wurde der geschätzte durch den gemessenen Umfang (in Zentimetern) geteilt und mit 100 multipliziert. Somit spiegeln Werte < 100 eine Unterschätzung und Werte > 100 eine Überschätzung der realen Körpermaße wider. Der Mittelwert aus BID-OA, BID-Taille und BID-OS ergibt den Gesamtindex (BID-Gesamt).

#### Evaluation der Gruppentherapie mithilfe des TBF-KBT-G

In Anschluss an den letzten Termin der körperpsychotherapeutischen Gruppentherapie füllten die Patientinnen den Therapiebeurteilungsfragebogen zur Konzentrativen Bewegungstherapie im Gruppen-Setting (TBF-KBT‑G; Seidler et al. 2018) aus. Der TBF-KBT‑G erfasst die wahrgenommenen Wirkungen und therapeutischen Faktoren der KBT-Gruppentherapie am Ende der Behandlung. Die in der vorliegenden Studie verwendete aktualisierte Version des TBF-KBT‑G besteht aus 18 Items, die 4 Subskalen zugeordnet werden: positive Erfahrungen mit der/m Therapeut/in (PE), positive Gruppenatmosphäre (PG), positive körperbezogene Erfahrungen und Effekte mit der KBT (PKEE) sowie therapeutischer Zugang zu sich selbst anhand der KBT (TZ). Die Antwortmöglichkeiten reichen von *trifft gar nicht zu* (0 Punkte) bis *trifft völlig zu* (4 Punkte). Die internen Konsistenzen der 4 Skalen sind mit Werten zwischen Cronbachs α = 0,75 und 0,90 akzeptabel bis sehr gut (Seidler et al. 2020).

### Statistische Analyse

Alle statistischen Analysen wurden mit IBM SPSS Statistics (Armonk, NY, USA) für Macintosh, Version 28 durchgeführt. Voraussetzungen für die jeweiligen Tests wurden geprüft (Normalverteilungsprüfung und Analyse der Ausreißer mithilfe des Quantil-Quantil[Q-Q]-Plots), und Spearman-Korrelationen verwendet.

Das Signifikanzniveau betrug α = 0,05. Veränderungen von Gewicht und Body-Mass-Index (BMI) wurden mithilfe von *t*-Tests bei Stichproben mit paarigen Werten untersucht. Veränderungen der Körpermaßschätzung wurden mithilfe einer „analysis of variance“ (ANOVA) mit Messwiederholungen mit den Faktoren Zeit (T1, T2) und Körperteil (OA, Taille, OS) untersucht sowie durch nach Holm korrigierte Post-hoc-*t*-Tests bei gepaarten Stichproben für die Körperteile ergänzt. Zur Bestimmung des Zusammenhangs von Therapiebeurteilung und Veränderung der Körpermaßschätzung wurden einseitige Spearman-Korrelationen mit den Subskalen des TBF-KBT‑G und der Differenz der BID-Indizes (Dif BID) zwischen den beiden Messzeitpunkten (T2 und T1) berechnet.

## Ergebnisse

### Stichprobe

Im Studienzeitraum wurden 76 Patientinnen stationär behandelt. Von diesen wurden 24 von der Studie ausgeschlossen. Es verließen 10 Patientinnen die Klinik vor Ende der Therapie. Neun Patientinnen nahmen an weniger als 8 Gruppenstunden teil und 3 nahmen nicht teil. Eine Patientin wurde aufgrund wiederholter Aufenthalte ausgeschlossen. Die endgültige Stichprobe umfasste 52 Patientinnen zwischen 12 und 18 Jahren (Mittelwert [M] = 15,35 Jahre, Standardabweichung [SD] ± 1,55 Jahre). Die Erkrankung begann knapp 11 Monate (M = 10,56 Monate, SD ± 11,86 Monate) vor der stationären Aufnahme, und die Patientinnen waren zum Krankheitsbeginn etwa 14 Jahre alt (M = 14,40 Jahre, SD ± 1,51 Jahre). Die meisten Patientinnen wiesen die restriktive Form der AN auf, und die Hälfte (*n* = 26) erhielt mindestens eine komorbide Diagnose (Tab. 1).

**Table 1:** 

	Patentinnen	
	Anzahl (*n*)	Anteil (%)
**Hauptdiagnose: Anorexia nervosa**		
F50.00 – Restriktiver Typ	37	71,15
F50.01 – Aktiver Typ	11	21,15
F50.1 – Atypisch	4	7,69
*Gesamt*	*52*	*100*
**Nebendiagnosen**		
F32 Depressive Episode	22	42,31
F34.1 Dysthymia	1	1,92
F40 Phobische Störungen	6	11,54
F42.1 Vorwiegend Zwangshandlungen (Zwangsrituale)	2	3,84
F60.6 Ängstliche (vermeidende) Persönlichkeitsstörung	1	1,92
F93.8 Sonstige emotionale Störung des Kindesalters	2	3,86
F81 Umschriebene Entwicklungsstörungen schulischer Fertigkeiten	1	1,92
X84.9 Absichtliche Selbstbeschädigung	11	21,15

### Psychopathologie

Die Ergebnisse der Symptomfragebogen sind in Tab. 2 aufgeführt.

**Table 2:** 

Fragebogen	M	± SD	Klinische Einordnung
*BDI‑2* (*n* = 48)	27,77	± 10,86	Mittelschwere Depression
*FFB* (*n* = 48)	130,77	± 37,53	Überdurchschnittliche Unzufriedenheit mit eigener Figur
*EDI‑C* (*n* = 24)			
Gesamt	216,42	± 56,03	Klinisch unauffällig^a^
Schlankheitsbestreben	24,54	± 9,30	Klinisch grenzwertig^a^
Bulimie	7,96	± 5,98	Klinisch unauffällig^a^
Unzufriedenheit mit dem Körper	30,71	± 11,14	Klinisch auffällig^a^
*EDE‑Q* (*n* = 21)	4,34	± 1,32	Klinisch auffällig

*BDI‑2* Beck-Depressions-Inventar, 2. Auflage, *EDE‑Q* Eating Disorder Examination – Questionnaire, *EDI‑C* Eating Disorder Inventory for Children, *FFB* Fragebogen zum Figurbewusstsein, *M* Mittelwert, *SD* Standardabweichung

^a^Einordnung nach Prozenträngen (*PR*) der weiblichen Kontrollgruppe: unauffällig (PR < 75); grenzwertig (PR ≥ 75 < 90); auffällig (PR ≥ 90) nach Kappel et al. (2012)

### Gewicht und Body-Mass-Index

Zwischen T1 und T2 nahmen die AN-Patientinnen mit durchschnittlich 8,72 kg signifikant zu (Gewicht T1: M = 42,74 kg, SD ± 5,02 kg; Gewicht T2: M = 51,46 kg, SD ± 4,78 kg; *t* (51) = 21,72, *p* < 0,001, *d* = 3,01). Der Standard Deviation Score (SDS) des BMI stieg ebenfalls signifikant um 1,76 Punkte (T1: M = −2,52 Punkte, SD ± 1,02 Punkte; T2: M = −0,75 Punkte, SD ± 0,44 Punkte; *t* (51) = 15,92, *p* < 0,001, *d* = 3,21).

### Körpermaßschätzung

**Figure 1:**
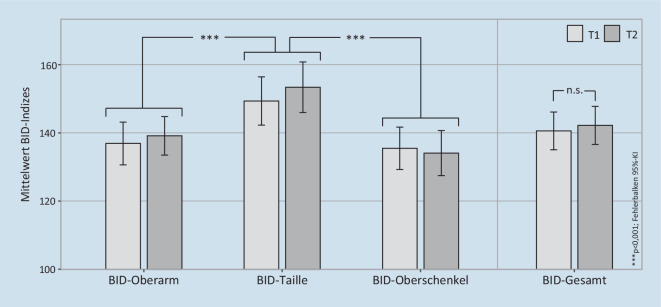

Die BID-T1-Erhebung fand etwa 10 Tage (M = 9,71 Tage, SD ± 6,47 Tage) nach der stationären Aufnahme statt und die BID-T2-Erhebung eine Woche (M = 7,54 Tage, SD ± 22,89 Tage) vor stationärer Entlassung. Die Dauer zwischen T1 und T2 betrug ca. 18 Wochen (M = 122,23 Tage, SD ± 50,93 Tage), und der stationäre Aufenthalt dauerte ca. 20 Wochen (M = 139,48 Tage; SD ± 56,93 Tage). Die Patientinnen wiesen eine Überschätzung der eigenen Körpermaße um ca. 40 % auf (Abb. 1). Dies veränderte sich nicht von T1 zu T2, Zeit: *F* (1,51) = 0,37, *p* = 0,547, *η*^*2*^ = 0,01. Die Körperteile wurden unterschiedlich stark überschätzt, Körperteil: *F* (2,102) = 23,14, *p* < 0,001, *η*^*2*^ = 0,31. Post-hoc-Tests zeigen an der Taille eine stärkere Überschätzung als an Arm und Oberschenkel, BID-Taille vs. BID-OA: *t* (51) = −4,72, *p*_*Holm*_ = 0,003, *d* = −0,66; BID-Taille vs. BID-OS: *t* (51) = −6,33, *p*_*Holm*_ = 0,003, *d* = −0,88, während Arm und Oberschenkel ähnlich stark überschätzt wurden, BID-OA vs. BID-OS: *t* (51) = 1,42, *p*_*Holm*_ = 0,162, *d* = 0,20. Die Interaktion Zeit × Körperteil zeigte keinen Effekt, *F* (2,102) = 1,67, *p* = 0,194, *η*^*2*^ = 0,03.

### Therapiebeurteilungsfragebogen zur Konzentrativen Bewegungstherapie im Gruppen-Setting

**Figure 2:**
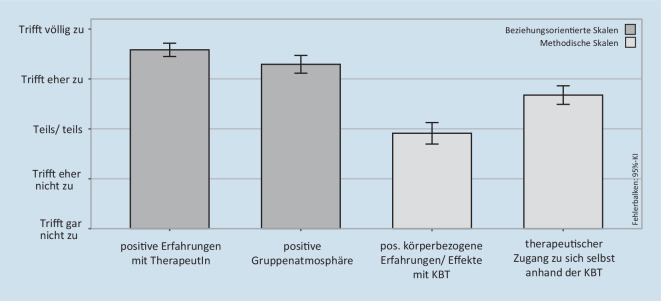

Die Patientinnen nahmen an 11,12 Gruppenstunden teil (SD ± 2,00 Gruppenstunden). Die Skalenmittelwerte von „positive Erfahrungen mit der/m Therapeut/in“ (PE; M = 3,59, SD ± 0,47) und „positive Gruppenatmosphäre“ (PG; M = 3,29, SD ± 0,63) lagen im Bereich trifft *eher zu* bis *trifft völlig zu*. Etwas geringer fielen die Mittelwerte der Skalen „positive körperbezogene Erfahrungen und Effekte“ (PKEE; M = 1,91; SD ± 0,76) und „therapeutischer Zugang zu sich selbst anhand der KBT“ (TZ; M = 2,68; SD ± 0,66) aus (Abb. 2).

### Zusammenhang zwischen BID und TBF-KBT-G

**Figure 3:**
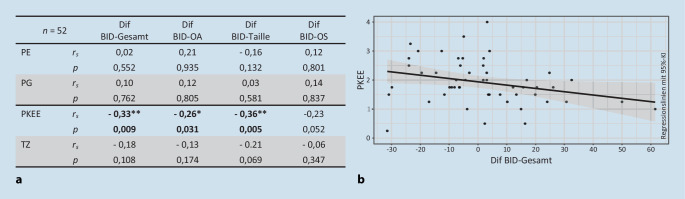

Die Skala PKEE zeigte eine moderate Korrelation mit der Veränderung der Körpermaßschätzung im Gesamtwert und an der Taille sowie eine schwache Korrelation mit der Veränderung am Oberarm (Abb. 3). Die Skalen PE, PG und TZ zeigen keine signifikante Korrelation.

## Diskussion

### Interpretation der Ergebnisse

Diese Arbeit stellt erste Ergebnisse zur Evaluation einer körperpsychotherapeutischen Gruppentherapie für jugendliche AN-Patientinnen auf Basis der KBT dar. Im Einklang mit der oben genannten ersten Hypothese berichten die Patientinnen im TBF-KBT‑G von positiven Erfahrungen mit der Therapeutin und dem Erleben einer positiven Gruppenatmosphäre. Dies kann als Voraussetzung verstanden werden, um positive Körpererfahrungen machen zu können. Die Patientinnen berichten darüber hinaus, über den KBT-Ansatz „teils/teils“ bis „eher“ einen tieferen Zugang zu sich selbst entwickelt zu haben. Teilweise gelang es den Patientinnen auch, positive körperbezogene Erfahrungen und Effekte mit der KBT zu erleben (Abb. 2). Angesichts des therapeutischen Fokus auf den aversiv erlebten Körper in einem von AN-Patientinnen oft als schwierig erlebten Gruppen-Setting können diese überwiegend positiven Beurteilungen als erfreuliches und ermutigendes Ergebnis beurteilt werden. Es zeigt, dass es gelingen kann, jugendliche AN-Patientinnen über eine verstärkte Selbstwahrnehmung zu einer Selbstöffnung und einem Austausch mit Peers über das häufig stark schambesetze Körpererleben zu ermutigen.

Im Vergleich mit einer Studie an erwachsenen PatientInnen verschiedener psychosomatischer und psychiatrischer Kliniken (Schreiber-Willnow 2022) wurden ähnliche Skalenmittelwerte für die beziehungsorientierten Skalen ermittelt, während die Mittelwerte der methodischen Skalen (PKEE, TZ) bei Erwachsenen vergleichsweise etwas höher ausfielen. Dies könnte auf eine etwas geringere Fähigkeit zur Nutzung der KBT-Methode durch jugendliche AN-Patientinnen hinweisen. Jedoch fehlen statistische Vergleiche, und es bleibt unklar, ob dies mit dem jugendlichen Alter oder dem Störungsbild der AN zusammenhängt. Insgesamt weisen die Ergebnisse auf ähnlich positive Erfahrungen mit der TherapeutIn und der Gruppenatmosphäre bei jugendlichen und erwachsenen PatientInnen hin und sprechen für die Anwendbarkeit von KBT-Gruppenangeboten im Jugendalter.

Übereinstimmend mit der zweiten oben genannten Hypothese war ein positives Erleben der Gruppentherapie, speziell das Erleben „positiver körperbezogener Erfahrungen und Effekte mit der KBT“, mit einer relativ besseren Körpermaßschätzung verbunden. Dies äußerte sich in einer positiven Korrelation mit der Veränderung des BID-Gesamtindex, und speziell mit den Indizes an Taille und Oberarm (Abb. 3). Aus dem Streudiagramm wird deutlich, dass Patientinnen, die mehr positive körperbezogene Erfahrungen machen konnten, ihre Körpermaße am Ende des Untersuchungszeitraums eher weniger überschätzten (negative Werte), während Patientinnen, denen dies schwerer fiel, sich eher noch mehr überschätzten (positive Werte)^1^. Im Gruppenmittel verringerte sich die Körpermaßschätzung allerdings nicht (Abb. 1), anders als in einer Vorläuferstudie (Dalhoff et al. 2019)^2^. Diese umfasste allerdings einen etwa 3 Wochen längeren Messzeitraum, in dem möglicherweise weitere therapeutische Fortschritte erzielt wurden. Fehlende Verbesserungen der Körpermaßschätzung könnten auch auf schwerere Krankheitsverläufe in der COVID-19-Pandemie zurückzuführen sein, während derer Essstörungssymptome zunahmen (Gilsbach et al. 2022), insbesondere bei AN-PatientInnen (Schlegl et al. 2020). Die beobachteten Zusammenhänge weisen auf relevante Unterschiede zwischen den Patientinnen in ihrem Ansprechen auf die körperpsychotherapeutische Gruppentherapie hin, wobei ein gelingendes Erleben positiver Körpererfahrungen mit einer Verbesserung der perzeptiven Komponente des Körperbildes – einem besonders hartnäckigen Symptom der AN (Boehm et al. 2016) – einherging.

### Limitationen und weiterer Forschungsbedarf

Die vorgestellten Studienergebnisse umfassen deskriptive Beschreibungen subjektiver Therapiebeurteilungen und keinen Wirksamkeitsnachweis im Sinne einer randomisierten kontrollierten Studie. Im aktuellen Studiendesign lassen sich die Einflüsse der verschiedenen therapeutischen Angebote im stationären Setting auf die Therapiebeurteilung sowie auf Veränderungen der Körpermaßschätzung nicht sicher der körperpsychotherapeutischen Gruppentherapie zuordnen. Die dargestellten Korrelationen sind auch angesichts der geringen Fallzahl und Effektstärken lediglich als erste, explorative Datenanalysen zu verstehen. Zudem wurde der TBF-KBT‑G für Erwachsene entwickelt und bisher nicht auf die Anwendbarkeit im Jugendalter überprüft. Nichtsdestotrotz werden die vorliegenden Ergebnisse als ermutigend beurteilt, und auf dieser Basis wird weitere Forschung zu Wirksamkeit und Wirkweise körperpsychotherapeutischer Behandlungsangebote für jugendliche AN-Patientinnen als erfolgversprechend angesehen. Wünschenswert sind randomisierte kontrollierte Studien zum Wirksamkeitsnachweis, z. B. ein Vergleich zwischen einem körperpsychotherapeutischen und einem anderen Gruppenangebot zusätzlich zur üblichen Behandlung. Des Weiteren empfehlen sich Langzeitstudien über Persistenz und Veränderung der perzeptiven Komponenten von Körperbildstörungen (Körpermaßschätzung, taktile und propriozeptive Körperwahrnehmung, körperbezogene Dissoziation und Fragmentierung).

## Fazit für die Praxis

- Verschiedene Komponenten der Körperbildstörung können mit unterschiedlichen Ansätzen gezielt behandelt werden.
- Jugendliche Anorexia-nervosa(AN)-Patientinnen weisen massive Störungen der Körperwahrnehmung mit Fragmentierung auf.
- Der körperpsychotherapeutische Ansatz auf Basis der Konzentrativen Bewegungstherapie (KBT) ermöglicht einen Zugang zum körperlichen Erleben durch konzentratives Bewegen und Wahrnehmen.
- Reflexion in der Gruppe hilft, Zusammenhänge von Körpererleben und anorektischer Symptomatik zu verstehen, und stärkt Zugehörigkeitsgefühl und Wohlbefinden.
- Trotz des Fokus auf den aversiv erlebten Körper berichten Patientinnen von positiver Beziehung zur Therapeutin, positiver Gruppenatmosphäre sowie teils von positiven körperbezogenen Erfahrungen und vertieftem Selbstzugang.
- Die Körpermaßüberschätzung besteht nach der Gewichtsrehabilitation fort, wobei positive körperbezogene Erfahrungen mit einer besseren Körpermaßschätzung verbunden waren.
- Die körperpsychotherapeutische Gruppentherapie ist eine sinnvolle Ergänzung zu bisherigen Ansätzen.

## Footnotes

1 Auch gesunde Kontrollprobandinnen überschätzen im Mittel ihre eigenen Körpermaße, dieser Effekt ist jedoch bei AN-Patientinnen deutlich stärker ausgeprägt. Ein Vergleich der aktuellen (teils überlappenden) Stichprobe mit der Kontrollgruppe aus Dalhoff et al. (2019) bestätigt eine signifikant erhöhte Überschätzung der eigenen Körpermaße auch in der hier untersuchen AN-Gruppe: ANOVA mit den Faktoren Gruppe (AN [*n* = 52], KG [*n* = 48]) und Körperteil (Oberarm, Taille, Oberschenkel), Haupteffekt Gruppe *F* (98, 1) = 83,47, *p* < 0,001, *η*^*2*^ = 0,46.

2 Die Patientinnen nahmen von T1 zu T2 sowohl bezüglich des gemessenen als auch des geschätzten Umfangs zu (Umfang geschätzt/gemessen: ANOVA mit den Faktoren Zeit [T1, T2] und Körperteil [Oberarm, Taille, Oberschenkel], Haupteffekt Zeit gemessen: *F* (1,51) = 261,78, *p* < 0,001, *η*^*2*^ *=* 0,84; Haupteffekt Zeit geschätzt: *F* (1,51) = 42,08, *p* < 0,001, *η*^*2*^ *=* 0,45).
